# Comprehensive Single‐Cell Characterization of LDL in the Ovarian Cancer Microenvironment and Its Prognostic Implications

**DOI:** 10.1155/mi/6540537

**Published:** 2025-12-22

**Authors:** Kang Tian, Jingjie Liu, Lei Zhou, Yue Gao, Shuzhen Wei

**Affiliations:** ^1^ Department of Oncology, The Affiliated Suqian Hospital of Xuzhou Medical University, Suqian, Jiangsu, China, xzmc.edu.cn; ^2^ Department of Respiratory Medicine, The Affiliated Suqian Hospital of Xuzhou Medical University, Suqian, Jiangsu, China, xzmc.edu.cn; ^3^ Department of Obstetrics and Gynecology, The Affiliated Huai’an Hospital of Xuzhou Medical University and The Second People’s Hospital of Huai’an, Huai’an, Jiangsu, China, xzmc.edu.cn; ^4^ Department of Oncology, Huai’an 82 Hospital, Huai’an, Jiangsu, China

**Keywords:** LDL, ovarian cancer, prognostic signature, single-cell RNA sequencing, tumor microenvironment

## Abstract

**Background:**

Low‐density lipoprotein (LDL) is a critical regulator of lipid metabolism and has been implicated in the development and progression of various malignancies. However, its specific roles and mechanisms in the ovarian cancer tumor microenvironment (TME) remain unclear. This study aimed to comprehensively elucidate the distribution, functional pathways, and prognostic value of LDL in ovarian cancer using single‐cell transcriptome analysis.

**Methods:**

Single‐cell transcriptome data from ovarian cancer patients were analyzed. The AUCell algorithm was used to score LDL‐related gene expression in different cell subsets, dividing cells into high and low LDL score groups. Functional pathway enrichment (Gene Set Variation Analysis [GSVA]) and cell–cell communication (CellChat) analyses were performed. Differentially expressed genes (DEGs) identified between the two groups were combined with bulk RNA‐seq data from eight cohorts to construct the LDL‐related ovarian cancer prognostic signature (LDLOCPS) using machine learning. Prognostic performance and immune landscape differences were evaluated between high and low LDLOCPS groups.

**Results:**

LDL was predominantly highly expressed in myeloid cells (macrophages and monocytes) and stromal cells (fibroblasts, smooth muscle cells, and endothelial cells) within the ovarian cancer TME. GSVA revealed that the high LDL score group was significantly enriched for pathways including epithelial‐mesenchymal transition (EMT), inflammatory response, coagulation, and angiogenesis. CellChat analysis demonstrated enhanced cell–cell communication involving IL6, CSF, and tenascin in the high LDL score group, with SPP1+ macrophages and monocytes showing stronger incoming and outgoing signals. The LDLOCPS model, constructed from bulk transcriptomic data and validated across eight cohorts, effectively stratified patients by risk; the high LDLOCPS group exhibited significantly worse overall survival. Receiver operating characteristic (ROC) and principal component analysis (PCA) analyses confirmed the robust predictive performance of LDLOCPS. Moreover, patients in the high LDLOCPS group showed reduced immune cell infiltration and lower expression of immune‐related genes, suggesting an immunosuppressive microenvironment.

**Conclusion:**

This study systematically reveals the spatial distribution of LDL within the ovarian cancer microenvironment and uncovers its regulatory roles in tumor progression through multiple signaling pathways. The LDLOCPS model provides a valuable tool for risk stratification and prognosis prediction in ovarian cancer. LDL‐mediated microenvironmental and immunosuppressive effects may offer novel insights for developing targeted and immunomodulatory therapies in ovarian cancer.

## 1. Introduction

Ovarian cancer ranks among the leading gynecological malignancies worldwide in both incidence and mortality rates [1]. According to global cancer statistics, there were ~310,000 new cases of ovarian cancer and more than 200,000 deaths worldwide in 2022 [2]. Due to the lack of specific symptoms and effective screening methods in the early stages, most ovarian cancer patients are diagnosed at an advanced stage, which profoundly affects treatment outcomes and prognosis [3]. At present, the standard treatment for ovarian cancer is cytoreductive surgery combined with platinum‐based chemotherapy. In recent years, the advent of targeted and immune therapies has benefited some patients, but the overall 5‐year survival rate remains less than 50%, far behind other gynecological tumors such as breast and endometrial cancer [4]. Therefore, elucidating the molecular mechanisms underlying ovarian cancer initiation, progression, metastasis, and resistance, and identifying novel therapeutic targets and prognostic biomarkers, has become a critical challenge in this field.

In recent years, the tumor microenvironment (TME) has attracted extensive attention as a crucial “soil” regulating tumor heterogeneity, progression, and therapeutic response [5–7]. The TME is composed not only of cancer cells, but also of immune cells (such as T cells, B cells, and macrophages), stromal cells (such as fibroblasts and endothelial cells), secreted factors, and extracellular matrix components [8]. The complex interplay and signaling networks among these components determine tumor biological behaviors and are involved in key processes such as immune evasion, angiogenesis, and drug resistance. In‐depth studies of the TME hold promise for the discovery of key regulatory molecules and mechanisms for ovarian cancer progression and provide a theoretical foundation for precision medicine and clinical translation. Aberrant lipid metabolism, especially disturbances in cholesterol and lipoprotein metabolism, has been shown to play important roles in various malignancies [9]. Low‐density lipoprotein (LDL), as the principal cholesterol transport carrier in the human body, delivers the raw materials essential for the synthesis of cellular membranes and signaling molecules via LDL receptor‐mediated endocytosis [10]. Recent studies have indicated that elevated LDL levels are closely associated with the development and progression of multiple cancers, including breast, colorectal, and prostate cancer [11]. LDL can promote tumor cell proliferation, migration, and invasion, influence tumor metabolic reprograming, and also affect the TME by regulating inflammatory responses and promoting epithelial‐mesenchymal transition (EMT) [12, 13]. However, the specific expression patterns of LDL in the ovarian cancer microenvironment, the signaling pathways it regulates, and its impact on immune cell infiltration, stromal remodeling, and cell–cell communication networks remain largely unexplored, and the underlying mechanisms are still poorly understood.

With the rapid development of single‐cell RNA sequencing (scRNA‐seq) and other multi‐omics technologies [14–17], researchers can now resolve the distribution, states, and functions of different cell subsets within tumor tissues at single‐cell resolution, and explore the transcriptional characteristics and interaction networks among various cells in the microenvironment [18]. This provides a new perspective for uncovering the heterogeneity and dynamic changes in the ovarian cancer TME and offers opportunities for identifying novel therapeutic targets and molecular markers.

Based on the limitations of existing research and the opportunities brought by technological advances, this study systematically analyzes the expression patterns of LDL in different cellular subsets within ovarian tumors using single‐cell transcriptome data. Furthermore, functional enrichment and cell–cell communication analyses were employed to elucidate the potential signaling pathways and regulatory networks mediated by LDL. By integrating multi‐center bulk RNA‐seq cohorts, we constructed an LDL‐related prognostic risk model for ovarian cancer using machine learning approaches, and comprehensively evaluated its clinical significance and association with immune features. Through this study, we aim to clarify the role and mechanisms of LDL in the ovarian cancer microenvironment and its influence on tumor progression and immune response, thereby providing theoretical and practical reference for the development of lipid metabolism‐targeted and immunotherapy combination strategies.

## 2. Method

### 2.1. Assessment of LDL Pathway Activity at the Single‐Cell Level

Single‐cell RNA‐seq data of ovarian cancer tissues were retrieved from GEO (Accession: GSE217517) [19] and processed in R using Seurat (v4.2). Quality control excluded cells with fewer than 200 or more than 6000 detected genes, mitochondrial transcript fraction > 10%, or identified as doublets (DoubletFinder/Scrublet); genes detected in fewer than 3 cells were removed. Counts were normalized with LogNormalize (scale.factor = 1e4), highly variable genes were identified using FindVariableFeatures (method = “vst”, nFeatures = 2000), and data were scaled with ScaleData while regressing out library size and mitochondrial percentage. Dimensionality reduction was performed by principal component analysis (PCA), followed by two‐dimensional embedding with *t*‐SNE/UMAP for visualization and graph‐based unsupervised clustering (Louvain/Leiden) on the shared nearest neighbor graph. Cluster annotation was based on canonical marker expression and reference mapping, enabling identification of major cell types, including epithelial cells, endothelial cells, cancer‐associated fibroblasts (CAFs), macrophages, and natural killer (NK) cells. To assess LDL pathway activity, the LDL‐related gene set was curated from the official GSEA website (http://www.gsea-msigdb.org, Supporting Information 1: Table S1). The AUCell R package was used to calculate the area under the curve (AUC) scores for LDL pathway activity at the single‐cell level. Specifically, AUCell ranks all expressed genes in each cell and determines the enrichment of the LDL gene set within the highly expressed genes, resulting in a quantitative AUC score per cell [20]. Cells were then classified into LDL‐high and LDL‐low groups based on their AUC scores. The distribution of LDL pathway activity among different cell types was subsequently analyzed to reveal heterogeneity of LDL signaling within the TME.

### 2.2. Pathway Activity Scoring and Enrichment Analysis

scRNA‐seq data from ovarian cancer tissues were analyzed. GSVA (Gene Set Variation Analysis) was used to calculate pathway enrichment scores for each Hallmark gene set at the single‐cell level [21]. According to LDL pathway‐related analysis, cells were divided into high and low LDL pathway activity groups. The GSVA score distributions of the two groups were compared for each hallmark pathway, and *t*‐tests were used to identify pathways with significant differential enrichment between groups. Spearman correlation coefficients between the proportions of different cell types were calculated within the high, low LDL activity groups and in all samples, and were visualized with correlation heatmaps to reveal patterns among cell types. Finally, Ro/e (representation over expected) analysis was performed to compare the distribution differences of cell types between groups by calculating the ratio of the observed proportion to the expected proportion, thereby evaluating the enrichment or depletion of specific cell subpopulations.

### 2.3. Cell–Cell Communication Analysis

To explore the changes in cell–cell communication associated with LDL pathway activity, CellChat (v1.6.1) [22] was applied to single‐cell RNA‐seq data from ovarian cancer samples. After quality control and cell type annotation, samples were classified into high (LDL_high) and low (LDL_low) LDL pathway activity groups according to predefined pathway scoring criteria. For each group, the normalized gene expression matrix and cell type labels were used as input for CellChat analysis. CellChat was run with default parameters to infer significant ligand–receptor interactions and to quantify both the number and strength of intercellular communications in each group. The information flow of each signaling pathway was calculated to measure its overall contribution to the communication network. Relative and absolute information flow values were compared between LDL_high and LDL_low groups to identify signaling pathways that were differentially active. Additionally, network centrality analysis was performed to assess outgoing and incoming interaction strength for each cell type, thereby revealing key sender and receiver cell populations under different LDL pathway states. All visualizations were generated in R using the CellChat and ggplot2 packages, and included bar plots of signaling pathway information flow, dot plots for ligand–receptor pair counts, bar graphs for total interaction counts and strength, as well as scatter plots for network centrality.

### 2.4. Construction and Validation of the LDL‐Related Ovarian Cancer Prognostic Signature (LDLOCPS)

To construct a robust LDL pathway‐related prognostic signature for ovarian cancer, we systematically integrated eight publicly available bulk transcriptomic datasets with overall survival information: TCGA, GSE140082 [23], GSE14764 [24], GSE17260 [25], GSE26193 [26], GSE32062 [27], GSE49997 [28], and GSE63885 [29]. To minimize non‐biological variations resulting from different platforms, sequencing batches, or sources, batch effects were corrected using the ComBat function from the sva (surrogate variable analysis) R package [30]. Candidate prognostic genes were first identified based on single‐cell transcriptomic analysis. Specifically, differentially expressed genes (DEGs) between groups with high and low LDL pathway activity were screened. These DEGs were subjected to univariate Cox proportional hazards regression analysis across all bulk cohorts. Genes that showed statistically significant prognostic value (*p* < 0.05) in at least four independent datasets were retained as candidate genes for subsequent risk model construction. To build and optimize the prognostic model, we evaluated various machine learning algorithms and their combinations, including Lasso, ridge regression, elastic net, and partial least squares regression for Cox models (plsRcox). The TCGA cohort was used as the training set, while the other seven cohorts served as independent external validation sets. Each candidate algorithm was trained using the TCGA cohort and assessed by the concordance index (c‐index) in all validation cohorts. The combination with the highest average c‐index across all datasets (Lasso plus plsRcox) was selected as the final method for constructing the LDLOCPS model. For each patient, risk scores were calculated based on gene coefficients derived from the established model. Patients were divided into high‐ and low‐risk groups by the median risk score within each cohort. Kaplan–Meier survival analysis and log‐rank tests were conducted to compare overall survival between groups, and the prognostic performance of the LDLOCPS was assessed in both the training and all seven validation cohorts.

### 2.5. Analysis of LDLOCPS Model in Relation to Immune‐Related Molecules and Immune Cell Infiltration

Immune‐related genes, including components of the major histocompatibility complex (MHC) class I and II, co‐stimulatory and co‐inhibitory molecules, were extracted from the RNA‐seq expression matrix of ovarian cancer samples. Differential expression analysis between high‐ and low‐LDLOCPS score groups was performed using the limma package in R. Multiple testing correction was conducted with the Benjamini–Hochberg method, and statistical significance was set at an adjusted *q*‐value of <0.05. The expression levels of immune‐related genes were visualized using heatmaps and violin plots. Tumor immune cell infiltration was quantified by applying CIBERSORT, TIMER, and ssGSEA algorithms to the RNA‐seq data to estimate the abundance of major immune cell subtypes, including T cells, B cells, and macrophages. Comparative analysis of immune cell infiltration between high‐ and low‐LDLOCPS groups was performed, with results visualized in boxplots or stacked bar charts. The correlations between core model gene expression and LDLOCPS risk score were evaluated using Pearson correlation analysis, with significance defined as an adjusted *q*‐value <0.05. Scatter plots were used to visualize the relationships between risk score and model gene expression.

### 2.6. Statistical Methods

All statistical analyses were performed using R software (version 4.2.0). Continuous variables were compared using the Student’s *t*‐test or Mann–Whitney U test as appropriate. Categorical variables were analyzed using the chi‐square test or fisher’s exact test. For comparisons among more than two groups, one‐way ANOVA or the Kruskal–Wallis test was applied. Kaplan–Meier survival analysis with log‐rank test was used to assess differences in overall survival. The prognostic value of risk models was evaluated by the concordance index (c‐index) and time‐dependent receiver operating characteristic (ROC) curves. Correlation analyses were conducted using Pearson or Spearman correlation coefficients as appropriate. All statistical tests were two‐sided, and a *p*‐value or adjusted *q*‐value <0.05 was considered statistically significant.

## 3. Result

### 3.1. Single‐Cell Transcriptomic Profiling Reveals Heterogeneous Activation of the LDL Pathway

To systematically elucidate the heterogeneity of LDL‐related pathway expression and its potential biological significance in the ovarian cancer TME, we first performed an in‐depth analysis of the GSE217517 scRNA‐seq dataset. Given the remarkable cellular heterogeneity within ovarian cancer tissues, accurately identifying and annotating the major cell populations is fundamental to clarifying the role of metabolic pathways. Thus, we visualized single‐cell data using tSNE plots based on the expression of classical marker genes, including EPCAM, PECAM1, VWF, COL1A1, ACTA2, LYZ, CD68, NKG7, NCR1, MS4A1, CD79A, and CD3E. These markers were shown to be selectively and specifically expressed in distinct clusters, effectively distinguishing epithelial, endothelial, fibroblastic, and immune cell populations (Figure 1A). Next, unsupervised clustering using the Seurat package identified 16 coherent cellular clusters (Figure 1B). Based on canonical marker expression, these clusters were further annotated as key cell types such as epithelial cells, endothelial cells, CAFs, monocytes, smooth muscle cells, NK cells, SPP1+ macrophages, proliferating cells, plasma cells, and dendritic cells (DCs) (Figure 1C). This refined clustering and annotation strategy comprehensively reflected the cellular complexity of ovarian cancer tissues and provided a robust foundation for downstream analyses. To further explore the activity of the LDL‐related pathway across cell populations, we applied the AUCell algorithm to quantify the enrichment of the LDL gene set at single‐cell resolution. The results revealed marked differences in LDL pathway activity among various cell subpopulations. Specifically, cells could be stratified into high‐ and low‐LDL activity groups according to their AUCell scores (Figure 1D). Spatial mapping of LDL activity demonstrated that epithelial cells, CAFs, and SPP1+ macrophages exhibited markedly higher LDL pathway activity than other clusters, whereas cell types such as endothelial cells and DCs showed relatively lower activity (Figure 1E). These findings indicate that activation of the LDL metabolic pathway is highly cell type‐specific, with especially prominent activity in tumor epithelial cells and critical stromal and immune populations within the TME. Together, these single‐cell findings reveal heterogeneous distribution of LDL pathway activity across ovarian cancer cell populations. This suggests a potential regulatory role for LDL metabolism not only in malignant cells but also in the tumor stromal and immune compartments, providing a solid basis for mechanistic studies of LDL involvement in tumor progression and the development of potential clinical interventions targeting this pathway.

Figure 1Single‐cell landscape and LDL pathway activity in ovarian cancer. (A) *t*‐SNE visualization of representative marker genes—EPCAM, PECAM1, VWF, COL1A1, ACTA2, LYZ, CD68, NKG7, NCR1, MS4A1, CD79A, CD3E—across all cells, delineating major populations. (B) Unsupervised clustering of the GSE217517 dataset resolved 16 discrete cell clusters. (C) Cluster annotation by canonical markers identified key cell types, including epithelial cells, endothelial cells, cancer‐associated fibroblasts (CAFs), monocytes, smooth muscle cells, natural killer (NK) cells, SPP1+ macrophages, proliferating cells, plasma cells, and dendritic cells (DCs). (D) Cells were stratified into LDL‐high and LDL‐low groups based on AUCell‐derived LDL pathway activity scores. (E) Distribution of LDL pathway activity (AUC) across cell types, highlighting intra–tumor microenvironment heterogeneity of LDL signaling.

**Figure:**
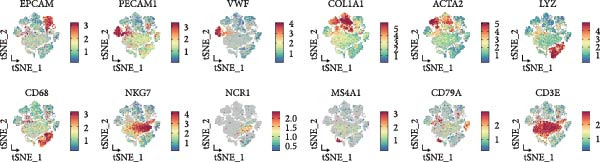
(A)

**Figure:**
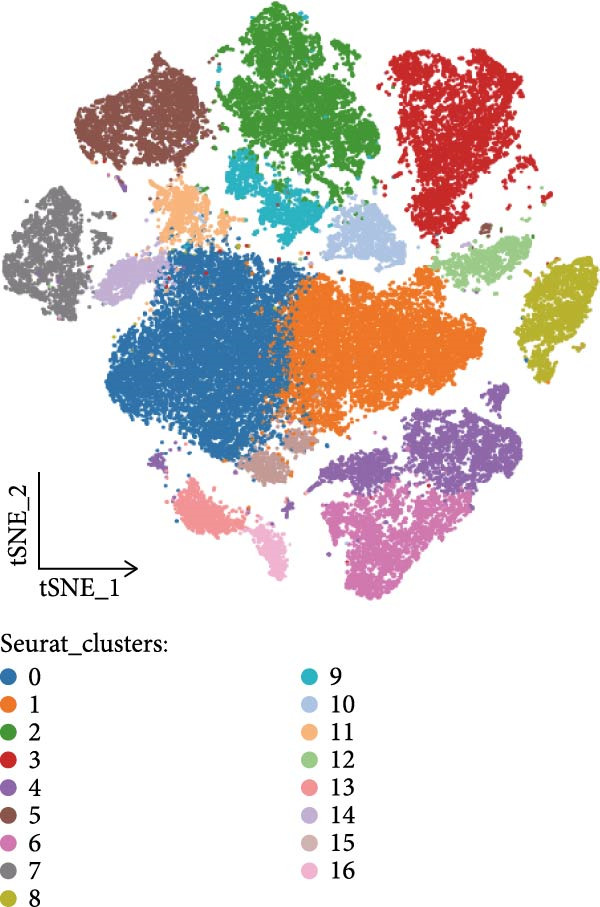
(B)

**Figure:**
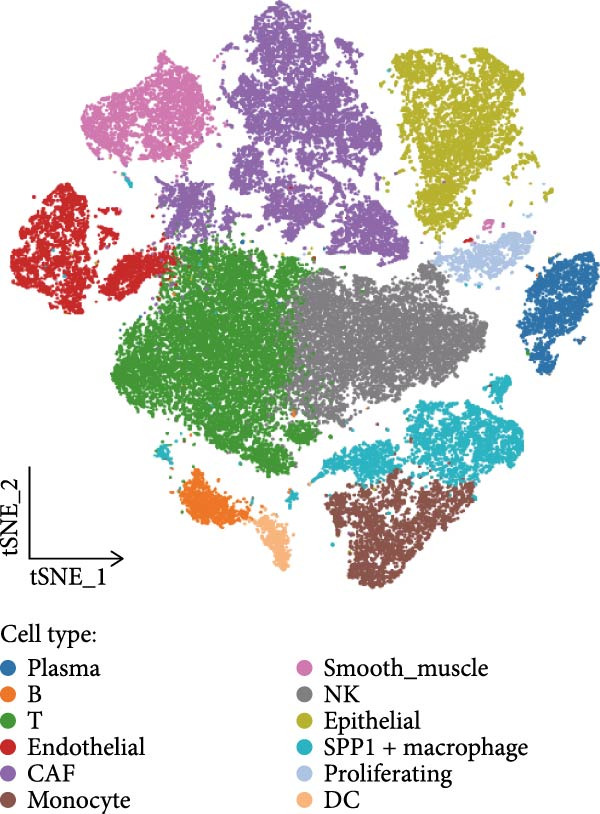
(C)

**Figure:**
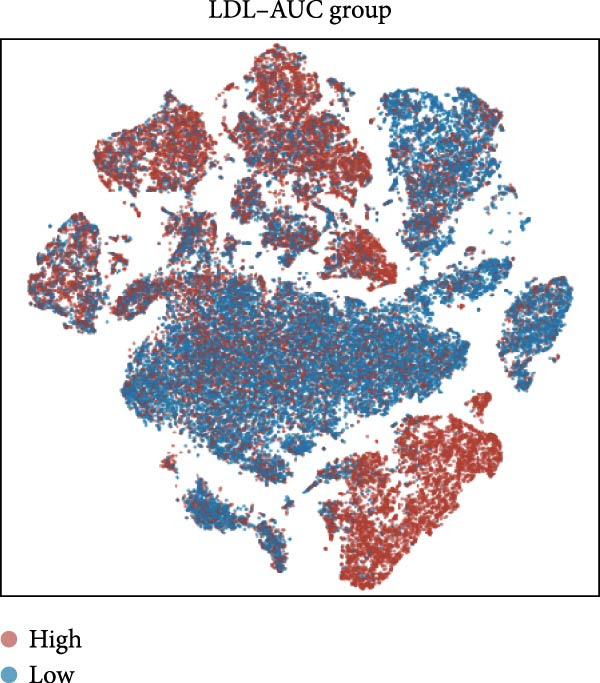
(D)

**Figure:**
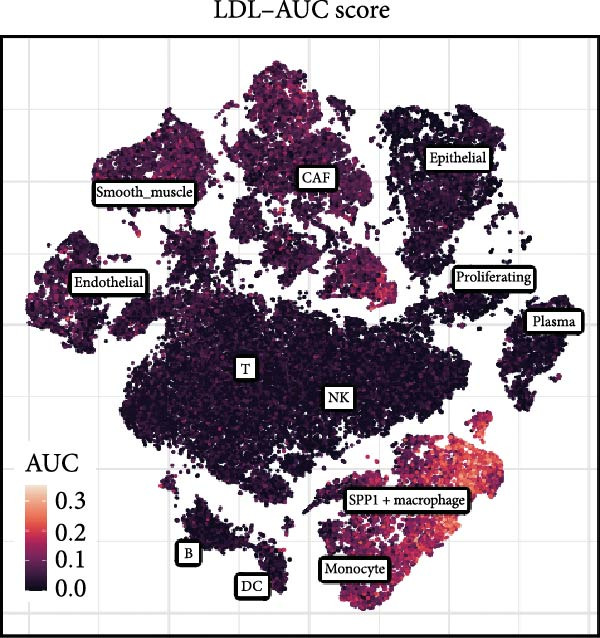
(E)

### 3.2. LDL Pathway Activity is Associated With Features of the TME

To investigate the biological processes associated with LDL pathway activity at the single‐cell level in ovarian cancer, we first performed hallmark pathway enrichment analysis comparing cells with high and low LDL pathway activity (Figure 2A). Cells with high LDL pathway activity were significantly enriched in pathways related to EMT, inflammatory response, coagulation, and angiogenesis, suggesting that LDL signaling upregulation is closely associated with tumor progression, immune regulation, and microenvironmental remodeling. Next, we analyzed the correlation patterns among major cell populations within the TME under different LDL pathway activity states. In the low LDL activity group (Figure 2B), immune cells such as T cells, B cells, and NK cells demonstrated strong positive correlations with each other, while exhibiting negative correlations with myeloid populations including monocytes and SPP1+ macrophages. Positive correlations were also observed between epithelial cells and CAFs. In contrast, the LDL high activity group (Figure 2C) displayed a distinct correlation network: the strong positive correlations among immune cells were preserved, but the positive correlation between monocytes and SPP1+ macrophages was further enhanced, indicating tighter interactions within myeloid populations. The overall sample analysis (Figure 2D) showed that cell types belonging to the same lineage (immune, myeloid, or structural) were usually positively correlated, whereas correlations across different lineages were predominantly negative. Finally, we performed Ro/e analysis to quantify differences in cell type enrichment between tissues with high and low LDL pathway activity (Figure 2E). We found that monocytes and SPP1+ macrophages were significantly enriched in tissues with high LDL pathway activity, whereas NK cells and proliferating cells were enriched in the low LDL activity group. These findings indicate that enhanced LDL signaling is accompanied by a shift in the TME towards a myeloid cell‐dominated landscape, while low LDL activity is associated with the prevalence of NK cells and proliferating populations, reflecting distinct immune ecologies under different metabolic states.

Figure 2LDL pathway activity shapes the tumor microenvironment in ovarian cancer. (A) Hallmark pathway enrichment analysis comparing single cells with high and low LDL pathway activity. Enriched pathways are indicated, including those related to EMT, inflammatory response, and angiogenesis. (B–D) Correlation heatmaps showing the relationships among major cell types within the tumor microenvironment in ovarian cancer. (B) Correlations in the low LDL activity group; (C) correlations in the high LDL activity group; (D) correlations across all samples. Color indicates Spearman correlation, ranging from −1 (blue) to +1 (red). Significant correlations are marked with asterisks ( ^∗^ *p* < 0.05,  ^∗∗^ *p* < 0.01). (E) Ro/e (representation over expected) analysis comparing cell type enrichment between high and low LDL pathway activity tissues. Bar length represents the degree of enrichment. Cell types with Ro/e > 1 are regarded as enriched in the respective group.

**Figure:**
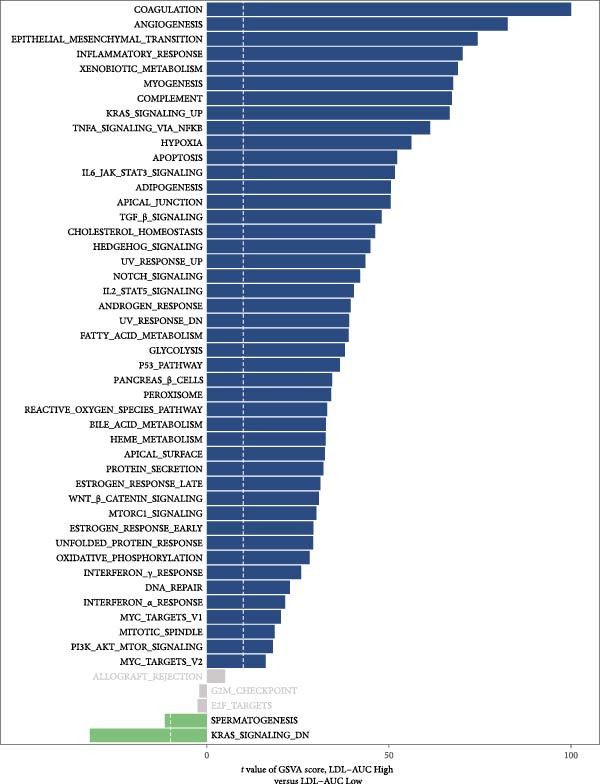
(A)

**Figure:**
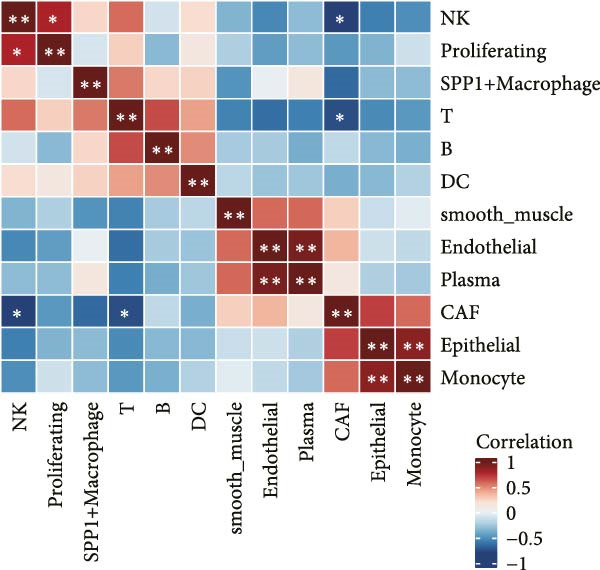
(B)

**Figure:**
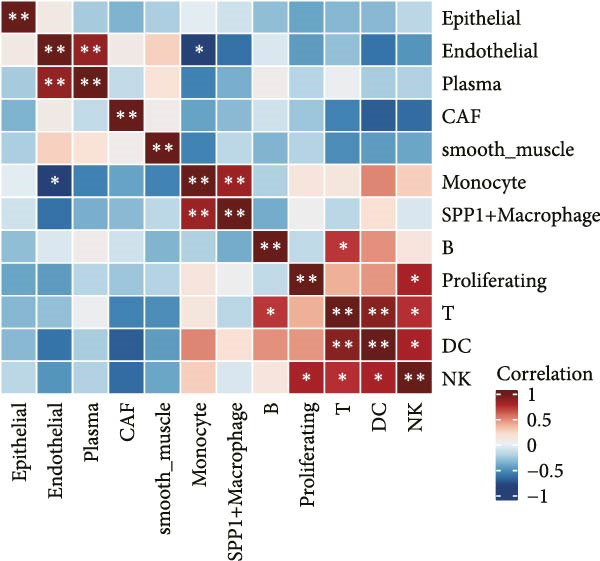
(C)

**Figure:**
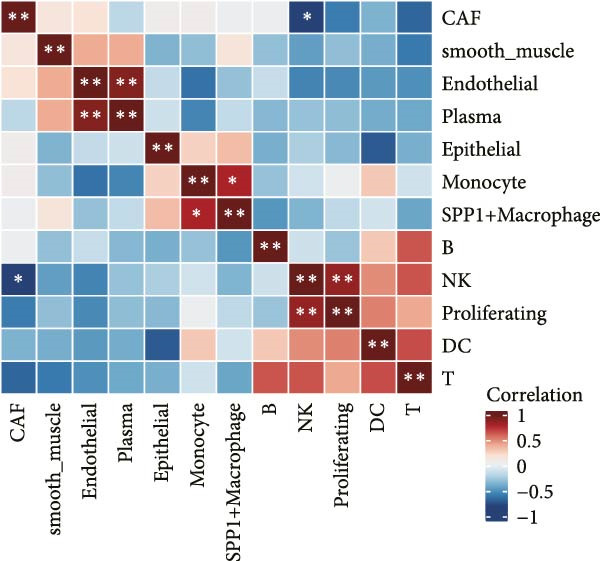
(D)

**Figure:**
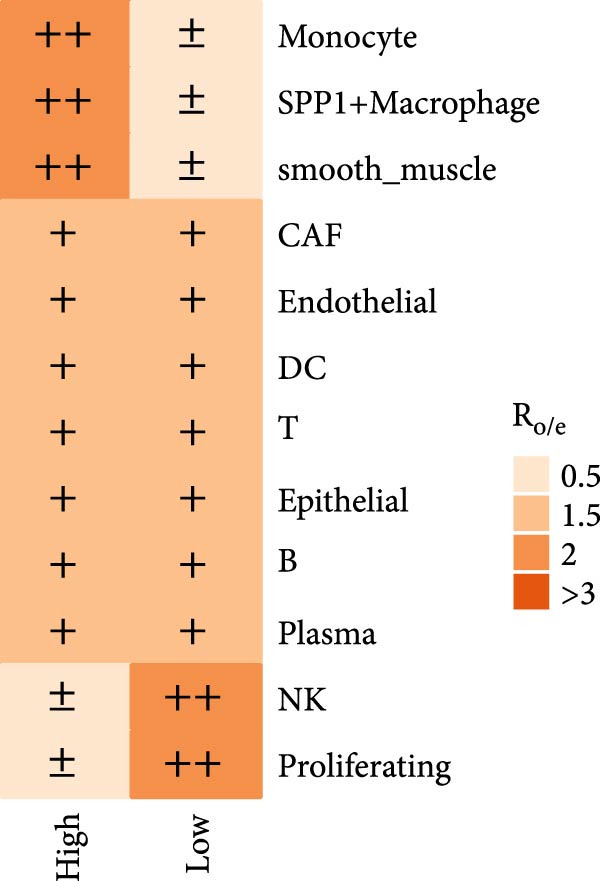
(E)

### 3.3. LDL Pathway Activity is Associated With Alterations in Cell–cell Communication

To further investigate the relationship between LDL pathway activity and cell–cell communication within the ovarian cancer microenvironment, we applied CellChat to compare signaling changes between samples with high and low LDL activity. As shown in Figure 3A,B, the information flow of the IL6, CSF, and TENASCIN signaling pathways was markedly increased in the high LDL group (LDL_high) relative to the low LDL group (LDL_low), indicating enrichment and enhancement of these pathways under elevated LDL activity. Notably, the functional consequences accompanying this enhancement have been reported in the literature: upregulated IL6 signaling activates the JAK/STAT3 cascade to promote tumor cell proliferation [31] and survival (e.g., increased MYC, Cyclin D1, and BCL2), induces EMT and invasiveness (e.g., elevated MMP2/9), and strengthens immunosuppression (e.g., increased PD‐L1 expression and Treg recruitment; impaired NK/CD8 effector function). CSF‐related signaling (CSF1/GM‐CSF/G‐CSF) has been reported to expand and immunosuppressively polarize myeloid populations—TAMs and MDSCs (e.g., increased ARG1, MRC1, and iNOS)—thereby dampening antigen presentation while promoting angiogenesis and matrix remodeling [32]. TENASCIN signaling (primarily Tenascin‐C, TNC) remodels the ECM and increases matrix stiffness via integrin–FAK/SRC and mechanotransduction pathways, activates YAP/TAZ, and consequently enhances tumor cell adhesion, migration, and immune exclusion [33]. A global comparison of intercellular communication (Figure 3C) further revealed that, although the LDL_low group exhibited a larger number of inferred interactions, the overall interaction strength was substantially higher in the LDL_high group. This suggests that elevated LDL activity not only redistributes signaling pathways but also intensifies signal transmission within specific pathways and may amplify the above pro‐tumor effects reported in prior studies.

Figure 3LDL pathway activity alters cell–cell communication networks in the ovarian cancer microenvironment. (A,B) CellChat analysis of outgoing signaling pathways between high (LDL_high, orange) and low (LDL_low, blue) LDL activity groups. (A) Relative information flow of each signaling pathway. IL6, CSF, and TENASCIN pathways are notably enriched in the LDL_high group. (B) Absolute information flow and the number of inferred ligand‐receptor pairs (dot size) for each pathway in both groups. (C) Comparison of overall cell–cell communication in the two groups. The LDL_low group shows a greater total number of inferred interactions, while the LDL_high group displays higher total interaction strength. (D,E) Network centrality analysis of cell types based on outgoing and incoming interaction strength. (D) In the LDL_low group, CAFs and smooth muscle cells are the main senders and receivers of signals. (E) In the LDL_high group, both outgoing and incoming interaction strength are markedly increased in SPP1+ macrophages and monocytes, indicating their enhanced roles in cell–cell communication under high LDL activity.

**Figure:**
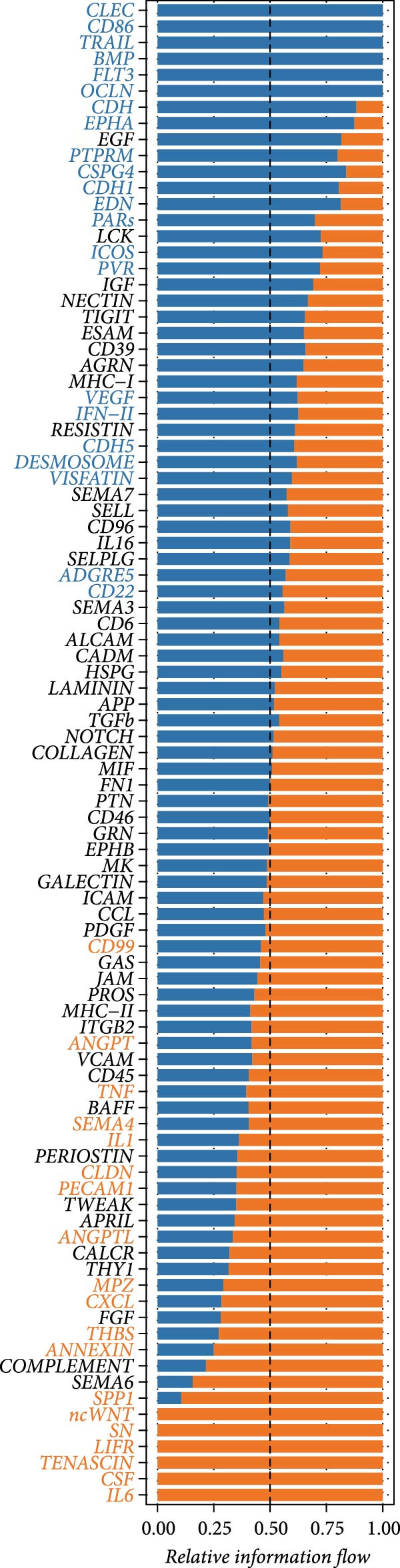
(A)

**Figure:**
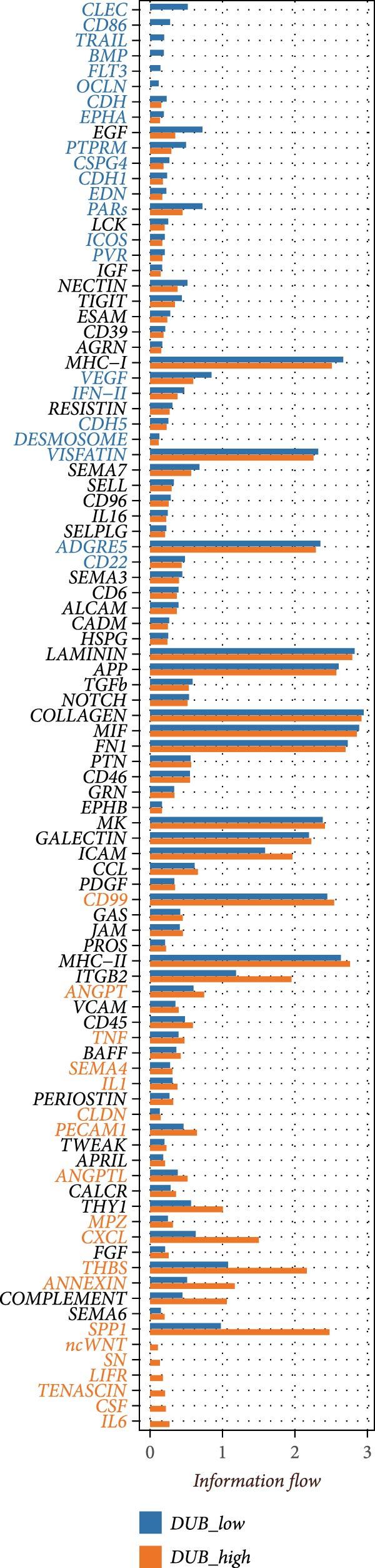
(B)

**Figure:**
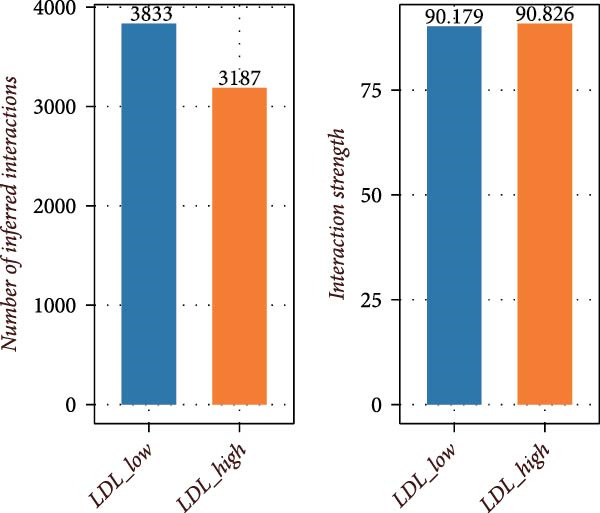
(C)

**Figure:**
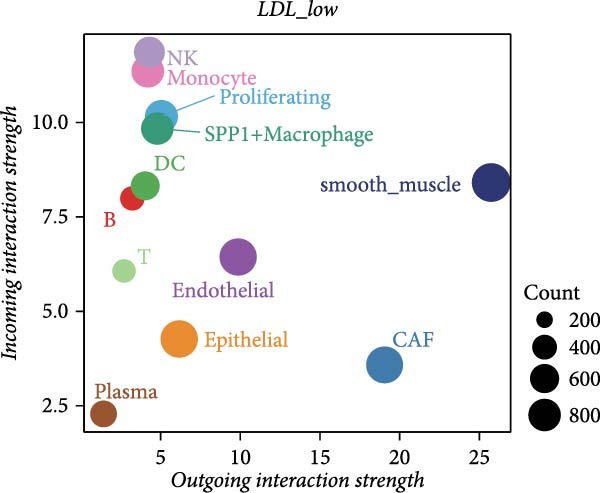
(D)

**Figure:**
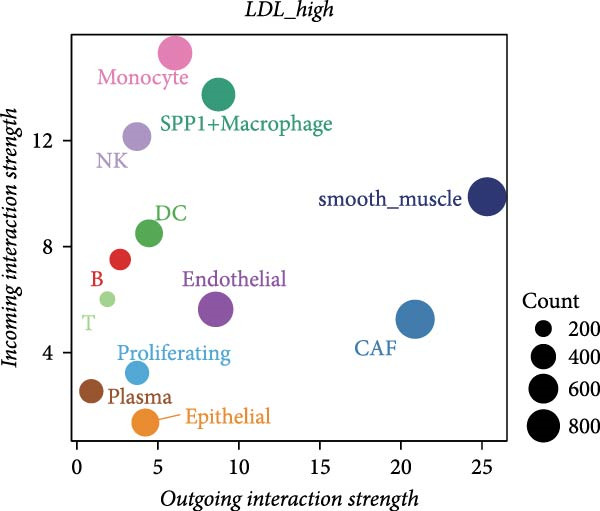
(E)

We also assessed the centrality and communication roles of major cell types under different LDL activity states. In the LDL_low group, CAFs and smooth muscle cells were the primary signal senders and receivers within the network (Figure 3D). In contrast, in the LDL_high group, SPP1+ macrophages and monocytes showed pronounced increases in both outgoing and incoming signaling strength (Figure 3E), indicating that these myeloid populations become more central hubs under high LDL conditions and cooperatively amplify the IL6, CSF, and TENASCIN axes to drive tumor growth, invasion, and immunosuppression. Collectively, these findings indicate that LDL pathway activity not only reshapes the strength and directionality of key signaling pathways in the TME but also, through enhanced interactions among specific immune cell populations and ECM mechanoreprogramming, promotes reorganization of immune regulatory networks and fosters tumor progression.

### 3.4. Robust Development and External Validation of the LDLOCPS

To establish a robust and generalizable LDL‐related prognostic model for ovarian cancer, termed the LDLOCPS, we systematically analyzed eight bulk transcriptomic datasets with available survival data (Figure 4A). DEGs between high and low LDL activity groups, identified by single‐cell analysis, were subjected to univariate Cox regression across all datasets. Only genes showing statistically significant prognostic value (*p* < 0.05) in at least four independent cohorts were retained, resulting in a 26‐gene candidate set encompassing both risk and protective factors (Figure 4B). For model construction, we explored various machine learning combinations, using TCGA as the training cohort and the other seven datasets as independent external validation cohorts. The prognostic performance of each approach was systematically compared based on the average concordance index (c‐index) across all cohorts. The Lasso combined with plsRcox algorithm achieved the highest mean c‐index and was selected for establishing the optimal LDLOCPS signature (Figure 4C). The resultant LDLOCPS risk model was then validated in all seven external cohorts. As shown in Figure 4D–J, the LDLOCPS model significantly stratified patients into high‐ and low‐risk groups with distinct overall survival in six out of seven validation datasets (log‐rank *p* < 0.05 for all except GSE63885). Similar prognostic segregation was observed in the TCGA training dataset (Supporting Information 2: Figure S1). Collectively, these results demonstrate that the LDLOCPS is a reliable and broadly applicable multigene prognostic tool, offering strong potential for risk stratification and individualized management of ovarian cancer patients across diverse populations.

Figure 4Development and validation of the LDL‐related ovarian cancer prognostic signature (LDLOCPS) across multiple cohorts. (A) Sample sizes of the eight bulk ovarian cancer transcriptomic datasets analyzed in this study. (B) Univariate Cox regression analysis of differentially expressed genes between high and low LDL pathway activity groups identified by single‐cell analysis. Only genes with statistically significant prognostic value (*p* < 0.05) in at least four cohorts were included, resulting in a 26‐gene candidate set. Genes with hazard ratios (HR) > 1 are classified as risk factors (orange); those with HR < 1 as protective factors (blue). (C) Comparison of multiple machine learning model combinations, using TCGA as the training cohort and the other seven datasets as external validation cohorts. The mean concordance index (c‐index) across all cohorts was used to identify the optimal model. The Lasso+plsRcox combination achieved the highest mean c‐index and was adopted as the final LDLOCPS model. (D–J) Kaplan–Meier survival curves demonstrating overall survival stratified by the LDLOCPS risk score in the seven external validation cohorts: GSE140082, GSE14764, GSE17260, GSE26193, GSE32062, GSE49997, and GSE63885.

**Figure:**
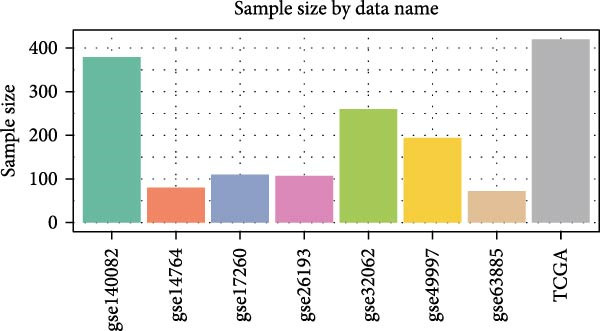
(A)

**Figure:**
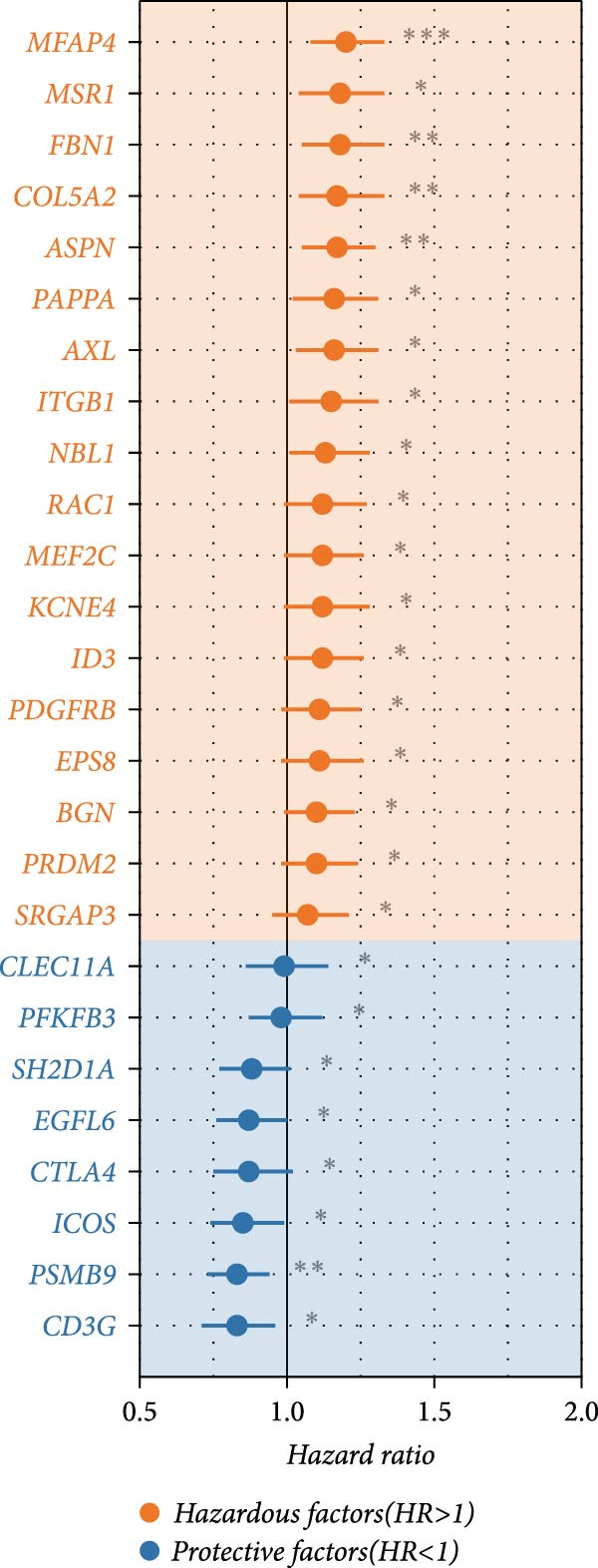
(B)

**Figure:**
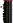
(C)

**Figure:**
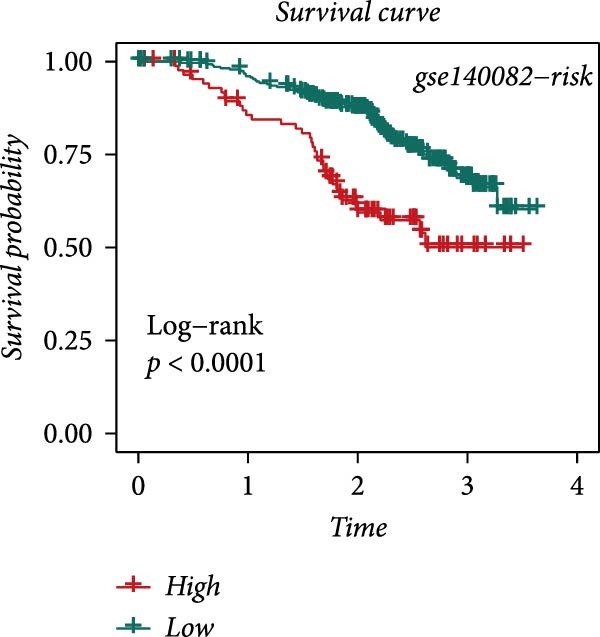
(D)

**Figure:**
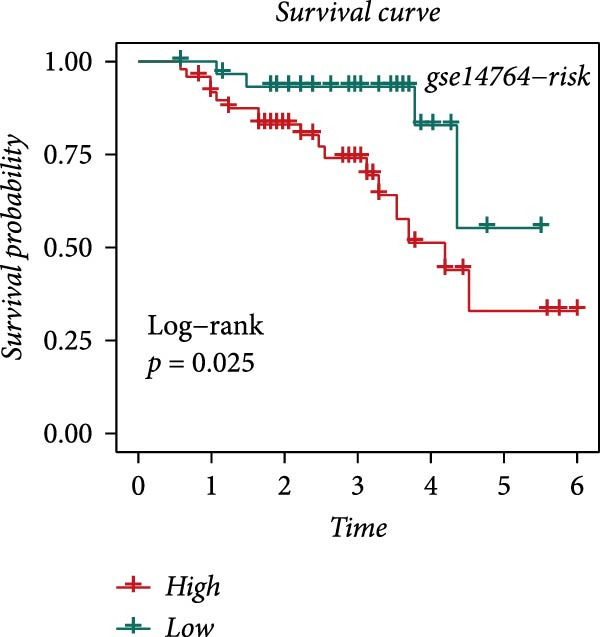
(E)

**Figure:**
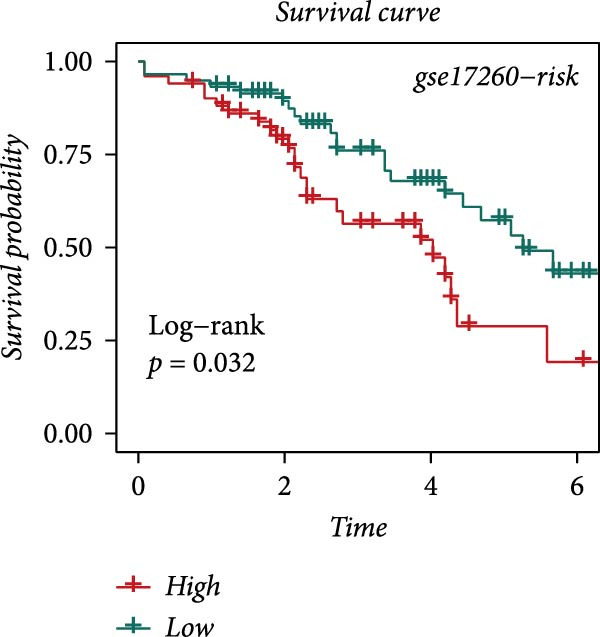
(F)

**Figure:**
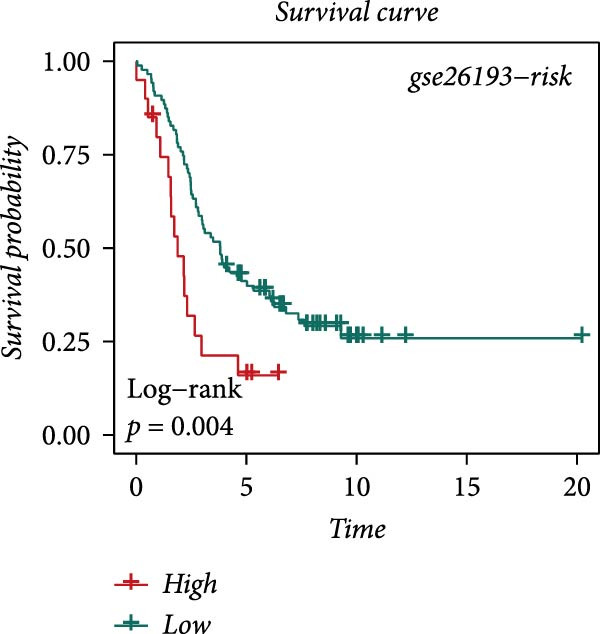
(G)

**Figure:**
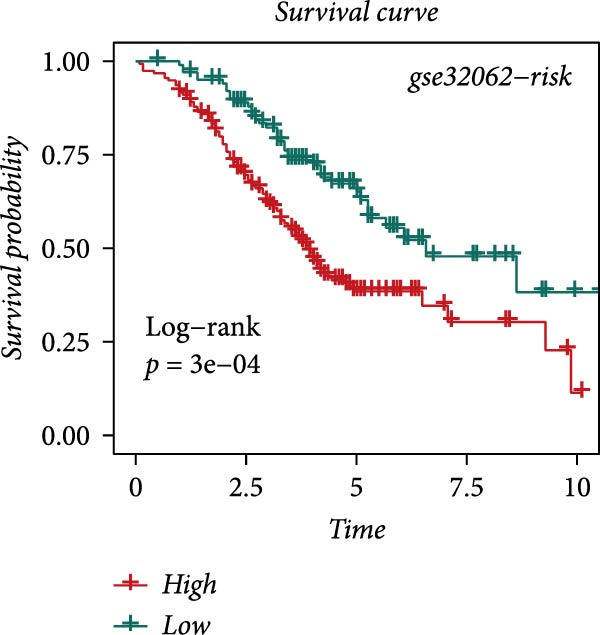
(H)

**Figure:**
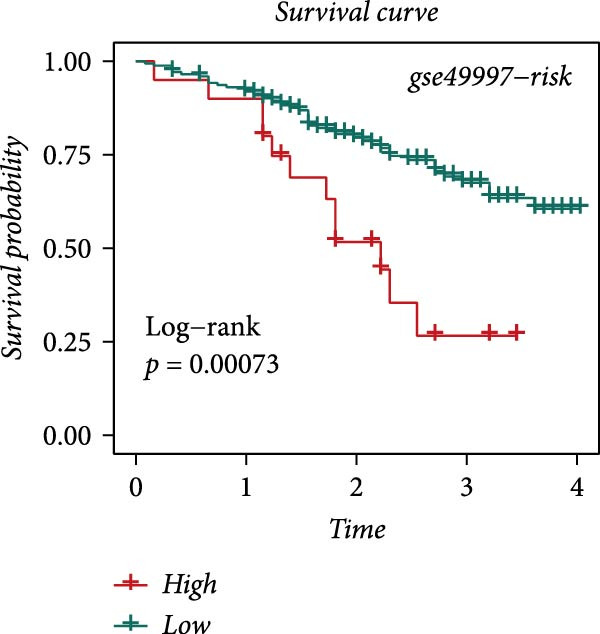
(I)

**Figure:**
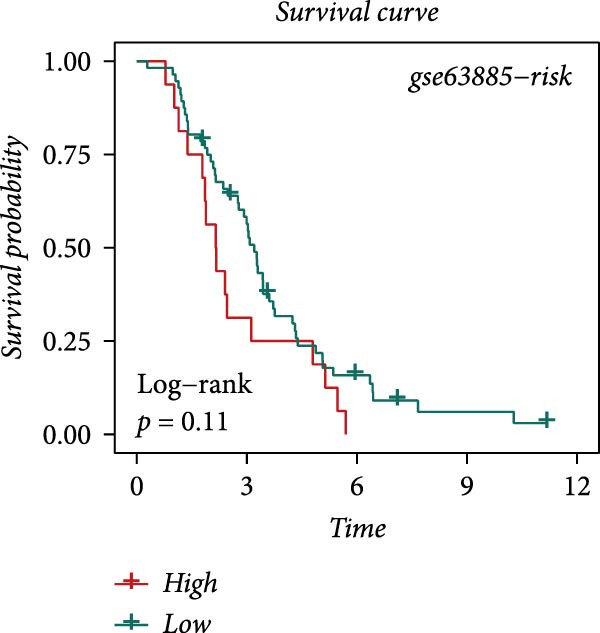
(J)

### 3.5. Assessment of the Accuracy and Discriminative Power of the LDLOCPS Model

The predictive performance of the LDLOCPS model was systematically evaluated across eight independent cohorts. As shown in Figure 5A, time‐dependent ROC curve analysis was conducted to assess the model’s ability to predict overall survival at 1, 3, and 5 years. The results demonstrated that the LDLOCPS model consistently achieved favorable AUC values at the 1‐year and 3‐year time points in both the training and external validation cohorts, with AUCs exceeding 0.7 in some datasets. These findings indicate that the LDLOCPS model possesses robust prognostic power and maintains its predictive accuracy across different populations. To further explore the discriminative capacity of the LDLOCPS model, PCA was performed based on the expression profiles of the genes included in the signature (Figure 5B). In all eight cohorts, high‐risk and low‐risk groups were clearly separated into two distinct clusters in the principal component space. This clear separation demonstrates that the LDLOCPS model not only predicts survival outcomes accurately but also effectively distinguishes between molecular subgroups of ovarian cancer samples. In summary, the LDLOCPS model exhibited high predictive accuracy and strong discriminative ability across multiple independent cohorts, further supporting its robustness and practical applicability for risk stratification in ovarian cancer.

Figure 5Evaluation of the predictive performance and discriminative power of the LDLOCPS model across eight ovarian cancer cohorts. (A) Time‐dependent ROC curves of the LDLOCPS risk score for predicting overall survival at 1, 3, and 5 years in eight independent cohorts. The area under the curve (AUC) values at each time point are shown within each plot, demonstrating the model’s prognostic accuracy in both training and validation datasets. (B) Principal component analysis (PCA) based on the expression profiles of genes included in the LDLOCPS model. Samples are categorized into high‐risk and low‐risk groups, which form clearly separated clusters in each cohort, highlighting the strong discriminative capacity of the LDLOCPS signature.

**Figure:**
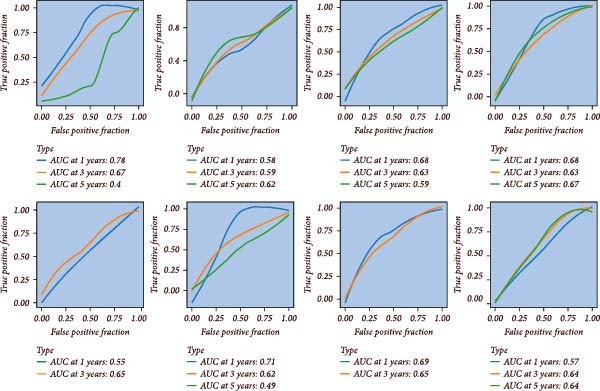
(A)

**Figure:**
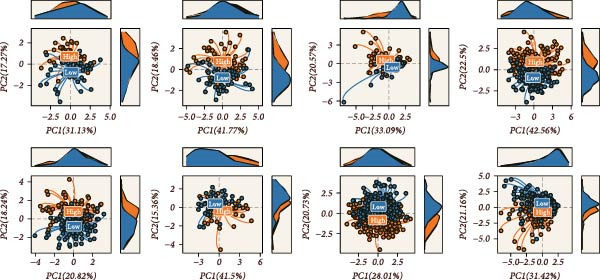
(B)

### 3.6. Association of the LDLOCPS Model With Immune Gene Expression, Immune Microenvironment, and Core Gene Correlations

First, the expression levels of immune‐related genes were compared between the high and low LDLOCPS groups (Figure 6A). The results revealed that the vast majority of immune‐related genes—including MHC class I, MHC class II molecules, and other antigen‐presenting molecules, as well as multiple immune co‐stimulatory and co‐inhibitory genes—were expressed at higher levels in the low LDLOCPS group, with some differences reaching statistical significance. This suggests that patients in the low LDLOCPS group may possess a more active antitumor immune response. Further analysis of the immune cell infiltration in the TME, conducted using multiple computational algorithms (Figure 6B), showed that the low LDLOCPS group exhibited significantly higher infiltration of most immune cell subtypes, such as T cells, B cells, and macrophages, as compared to the high LDLOCPS group. The differences were especially pronounced among key effector cells like T cells and macrophages. These findings are consistent with the higher expression of immune genes, highlighting an “immune‐enriched” TME in the low‐risk group. Correlation analyses between core model genes and the LDLOCPS risk score (Figure 6C–J) demonstrated that most genes (such as AXL, CD36, and ICOS) were positively correlated with the risk score. Notably, PSMB9 showed a significant negative correlation with the LDLOCPS score (*r* = −0.56, *q* = 0), indicating that PSMB9 expression is higher in the low‐risk, immune‐activated group. Considering its tight association with enhanced immune activity and favorable prognosis, PSMB9 may serve not only as a marker of better outcomes but also as a potential target for immunotherapy. In summary, ovarian cancer patients with low LDLOCPS scores are characterized by higher expression of immune‐related genes and greater immune cell infiltration, which reflects an immune‐activated, anti‐TME. PSMB9, as a core gene negatively correlated with the LDLOCPS score, may serve as a novel marker for immune phenotyping and a promising therapeutic target. These findings provide new insights into the relationship between the LDLOCPS model and the immune microenvironment, and inform future development of precise immunotherapeutic strategies.

Figure 6The relationship between the LDLOCPS model, immune gene expression, immune infiltration, and gene–risk score correlations in ovarian cancer. (A) Heatmap and violin plots showing differences in the expression of immune‐related genes (including MHC‐I, MHC‐II, other antigen presentation molecules, co‐inhibitors, and co‐stimulators) between high‐ and low‐LDLOCPS groups. Most immune genes are more highly expressed in the low‐LDLOCPS group. Statistical significance is indicated ( ^∗∗∗^ *p* < 0.001; ns, not significant). (B) Landscape of immune cell infiltration in high‐ and low‐LDLOCPS groups as assessed by multiple computational algorithms. The low‐LDLOCPS group shows markedly higher levels of immune cell infiltration, especially for T cells and B cells. (C–J) Correlation analyses of LDLOCPS risk score with representative model genes. Each scatter plot shows the relationship between risk score and gene expression (EGFL6, MSR1, PAPPA, PSMB9, AXL, CD36, ICOS, and MIFAP1); Pearson correlation coefficients (*r*) and adjusted *q*‐values are provided. Notably, PSMB9 is significantly negatively correlated with LDLOCPS score, indicating potential as an immunotherapeutic target. Orange and blue points denote high‐ and low‐LDLOCPS score groups, respectively. ^∗^ *p* < 0.05; ^∗∗^ *p* < 0.01.

**Figure:**
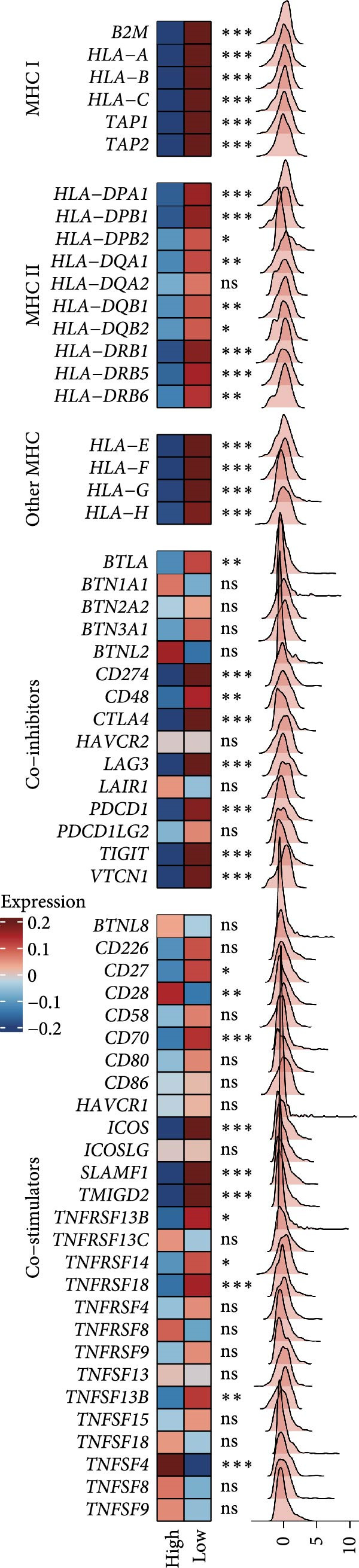
(A)

**Figure:**
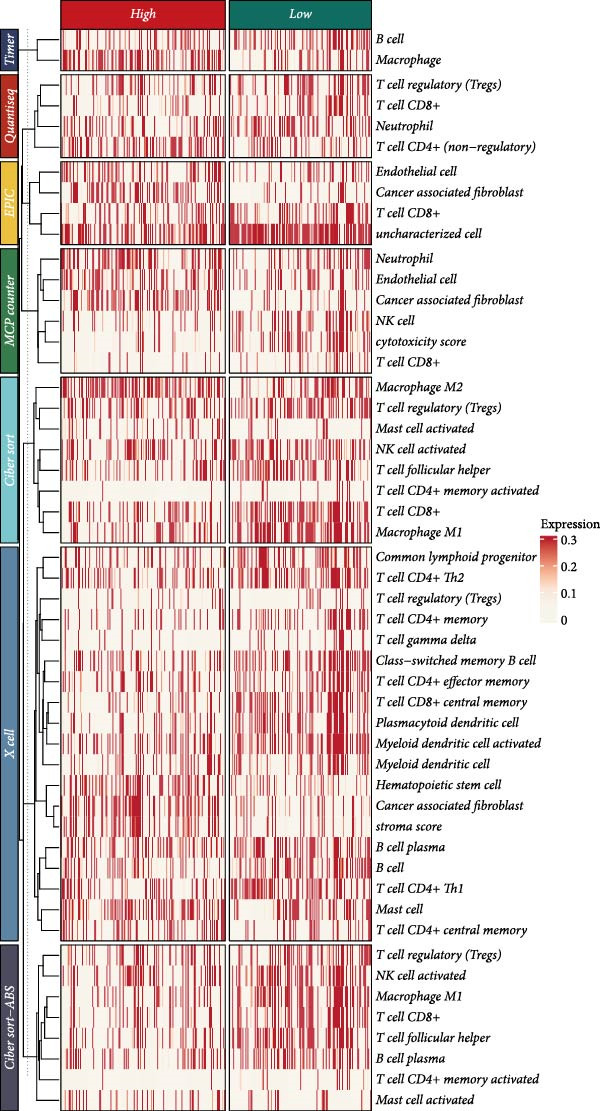
(B)

**Figure:**
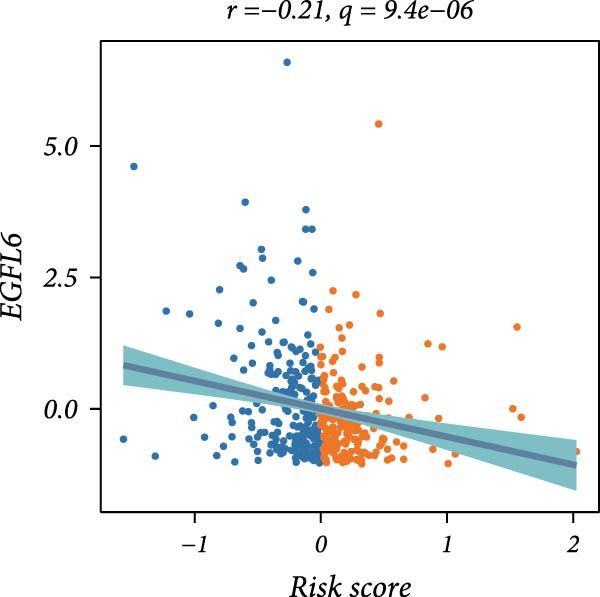
(C)

**Figure:**
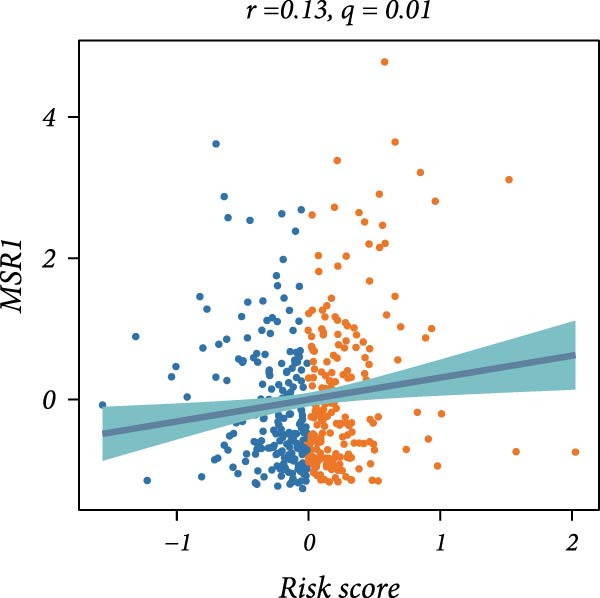
(D)

**Figure:**
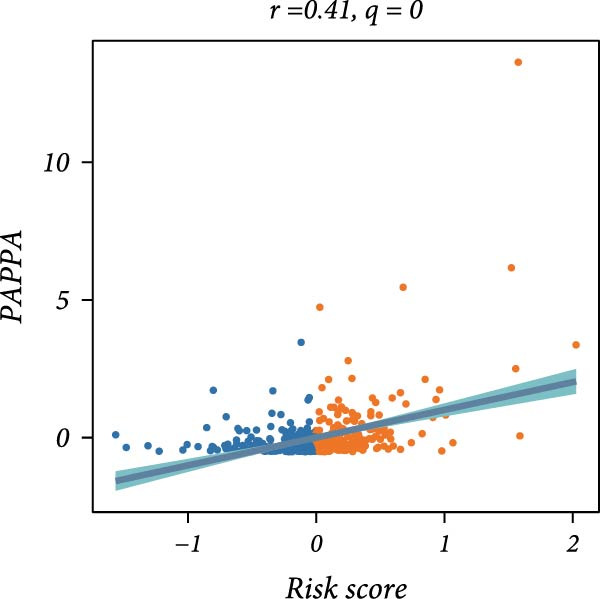
(E)

**Figure:**
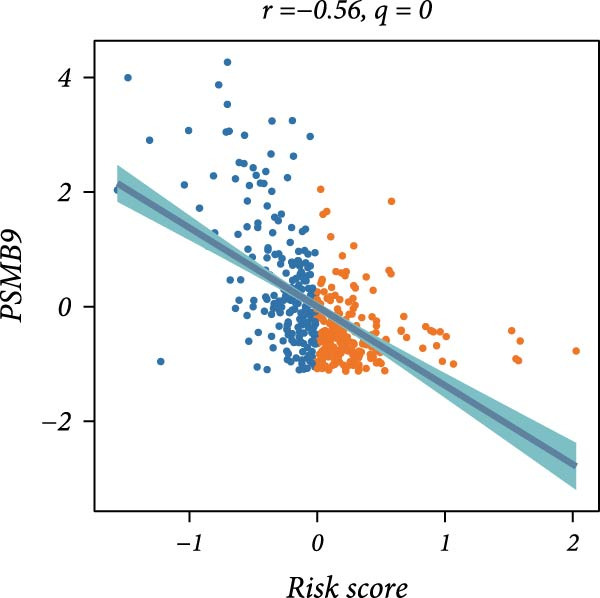
(F)

**Figure:**
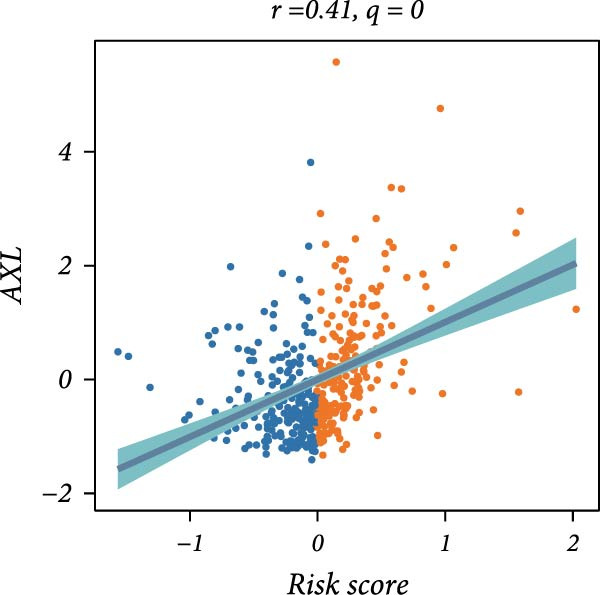
(G)

**Figure:**
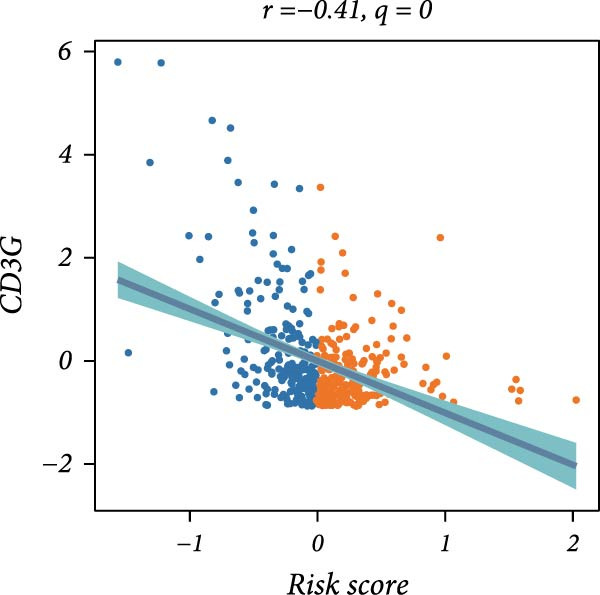
(H)

**Figure:**
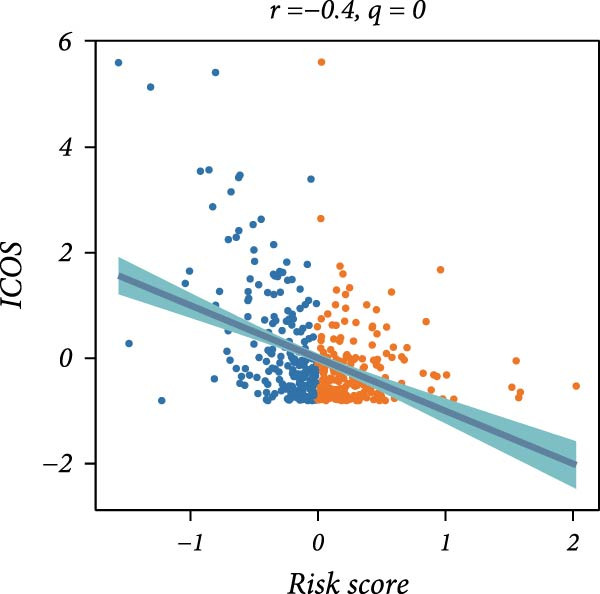
(I)

**Figure:**
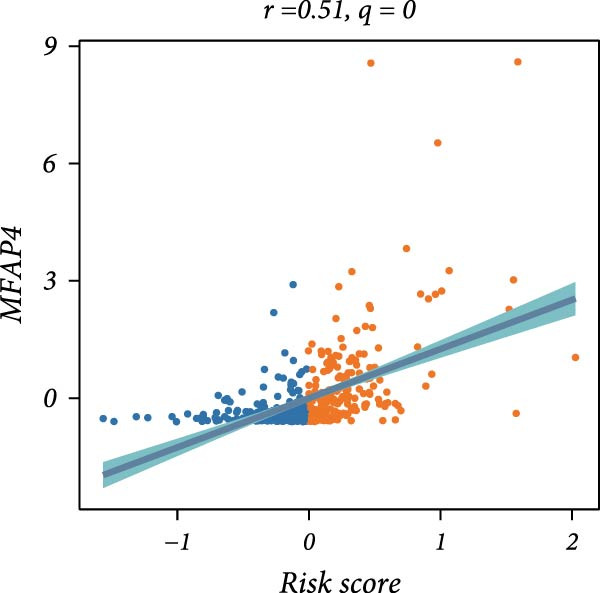
(J)

## 4. Discussion

In recent years, cancer metabolic reprograming has attracted increasing attention due to its essential roles in shaping the TME, influencing cellular heterogeneity, and determining patient prognosis [34, 35]. Numerous studies have suggested that lipid metabolism, especially the LDL pathway, is aberrantly activated in various solid tumors [36–38] and is closely associated with cancer cell proliferation, invasion, and immune regulation; however, the single‐cell expression heterogeneity and mechanisms of the LDL pathway in ovarian cancer remain unclear. In this study, we first systematically mapped the expression distribution of the LDL pathway across various cell populations of ovarian cancer at the single‐cell level. Traditionally, LDL pathway abnormalities have been considered mainly involved in tumor cell proliferation and metabolism, but our single‐cell analysis revealed that LDL signaling is significantly upregulated not only in malignant epithelial cells but also in key microenvironmental components such as CAFs and SPP1+ macrophages, while certain vascular endothelial cells and DCs exhibited low expression. These findings expand the understanding of lipid metabolism dysfunction in multicellular ecosystems and suggest that the LDL pathway may play a crucial role in dynamic, cross‐cell‐type regulation within the ovarian cancer microenvironment.

The composition and signaling functions of the TME are profoundly influenced by the metabolic state of the tumor; previous research has demonstrated that lipid metabolic remodeling can affect oncogenic processes such as inflammatory responses and EMT [39]. Our GSVA enrichment and correlation analyses showed that cells with high LDL pathway activity are mainly associated with biological processes including EMT, inflammation, coagulation, and angiogenesis, indicating their potential to promote tumor progression and microenvironmental remodeling. Meanwhile, under conditions of high LDL activity, myeloid immune cells (such as SPP1+ macrophages and monocytes) further strengthened their spatial and signaling connections, whereas NK cells and proliferating cells were more enriched in low LDL activity states. Cell–cell correlation results suggest that upregulation of the LDL pathway transforms the immune ecology from lymphocyte‐dominated to myeloid‐dominated, and facilitates the remodeling of interlineage cellular interaction networks. Aberrant intercellular communication is regarded as a critical molecular foundation for TME transformation and immune escape [40, 41]. Previous work has largely focused on changes in gene expression [42], while in this study, we further applied the CellChat algorithm to explore the relationship between LDL pathway activity and the reorganization of signaling networks. Our analysis demonstrated that in the high LDL activity group, oncogenic signaling pathways such as IL6, CSF, and TENASCIN exhibited notably enhanced information flow, with SPP1+ macrophages and monocytes serving as central hubs for signal output and reception. Both the overall communication strength and pathway number were superior to those in the low LDL group. These results indicate that enhanced LDL metabolism not only intensifies the activation of immune‐related pathways but also drives the formation of a more compact signaling network via myeloid cells, thereby accelerating the establishment of an immunosuppressive microenvironment.

Reliable and generalizable molecular prognostic models are pivotal in precision oncology [43, 44]. Extensive large‐cohort evidence indicates that single biomarkers rarely capture the complexity of tumor risk heterogeneity, making multigene risk signatures a preferred strategy. Accordingly, we integrated multiple large‐scale, independent cohorts, and rigorously filtered LDL‐related molecular features using single‐cell analyses combined with survival statistics to develop an LDL‐based prognostic scoring model, LDLOCPS. The model demonstrated stable and superior predictive performance and risk stratification across several external validation cohorts. Moreover, PCA revealed a clear molecular separation between high‐ and low‐risk groups, indicating that LDLOCPS not only captures underlying biological distinctions in ovarian cancer but also provides a robust tool for prognosis assessment and individualized therapeutic guidance.

Cancer metabolic abnormalities are closely coupled to the immune microenvironment, impacting immune gene expression and patterns of immune cell infiltration [45, 46]. Multiple recent studies have focused on immunotherapy patient selection and response prediction [47], but few have linked lipid metabolic risk signatures with immune subtypes. Our findings demonstrate that patients in the low‐risk LDLOCPS group display higher expression of antigen presentation and immune co‐stimulatory/inhibitory molecules, with significantly increased infiltration of key immune populations such as T cells and macrophages—representing an “immune‐activated” microenvironment. Meanwhile, core genes such as PSMB9 were specifically highly expressed in the LDLOCPS low‐risk group and negatively correlated with the risk score, supporting its potential as a marker for immune subtyping and as a therapeutic target. Together, these observations newly elucidate the three‐way synergy among lipid metabolism, immune regulation, and clinical prognosis.

This study has several limitations. First, all findings are derived from analyses of public single‐cell and bulk transcriptomic datasets, so the conclusions are associative rather than causal and lack direct experimental validation. Second, key mechanistic inferences—such as the linkage between LDL pathway activity, EMT/inflammation, and the role of core genes (e.g., PSMB9)—have not been confirmed at the protein or spatial levels (IHC/mIF/spatial‐omics) or via functional perturbation (loss/gain‐of‐function, rescue, coculture). Third, although the risk model was evaluated across multiple cohorts, potential batch effects, clinical heterogeneity, and follow‐up biases may limit generalizability; prospective, temporal‐external, and real‐world validations with calibration and decision‐curve analyses are needed. In summary, this study, for the first time using combined single‐cell and multi‐cohort approaches, reveals the multicellular heterogeneity, immune microenvironment remodeling, and prognostic significance of LDL metabolic dysregulation in ovarian cancer. The established LDLOCPS model provides a novel avenue for accurate risk stratification and individualized immunotherapy optimization.

## Ethics Statement

This study did not involve any animal or human experiments. All analyses were performed using publicly available datasets; therefore, no ethical approval was required.

## Disclosure

All authors have read, contributed to, and approved the final manuscript.

## Conflicts of Interest

The authors declare no conflicts of interest.

## Author Contributions

Kang Tian, Yue Gao, and Shuzhen Wei conceptualized the study design and oversaw its implementation. Jingjie Liu and Lei Zhou were responsible for data acquisition and curation. Kang Tian and Jingjie Liu performed statistical analyses and bioinformatic interpretations. Kang Tian, Jingjie Liu, and Lei Zhou drafted the initial manuscript. Yue Gao and Shuzhen Wei provided critical revision for intellectual content and granted final approval of the submitted version.

## Funding

This work was supported by the 2023 Suqian Sci and Tech Program (Grant Z2023108), the 2024 Suqian Sci and Tech Program (Grant Z2024100), and Young Elite Scientists Sponsorship Program by Jiangsu Association for Science and Technology (Grant JSTJ‐2024‐649).

## Supporting Information

Additional supporting information can be found online in the Supporting Information section.

## Supporting information


**Supporting Information 1** Table S1: Specific genes of the LDL‐related gene set.

**Supporting Information 2** Figure S1: Kaplan–Meier survival curve showing LDLOCPS performance in the TCGA training cohort.

## Data Availability

All datasets analyzed in this study are publicly available. The single‐cell RNA sequencing dataset used for ovarian cancer tissues was obtained from the GEO database (Accession: GSE217517). Bulk RNA‐seq datasets with overall survival information, including TCGA, GSE140082, GSE14764, GSE17260, GSE26193, GSE32062, GSE49997, and GSE63885, were retrieved from the GEO database (https://www.ncbi.nlm.nih.gov/geo/) and the TCGA data portal (https://portal.gdc.cancer.gov/). All data used in this study are available from the corresponding repositories, and no new datasets were generated. The processed data and scripts supporting the current findings are available from the corresponding author upon reasonable request.
