# Supplementary figures and images for: Comprehensive Single‐Cell Characterization of LDL in the Ovarian Cancer Microenvironment and Its Prognostic Implications
Source: Mediators Inflamm. 2025 Dec 22;2025:6540537. doi: 10.1155/mi/6540537 (PMC12767482; doi:10.1155/mi/6540537)

## ***Survival curve***

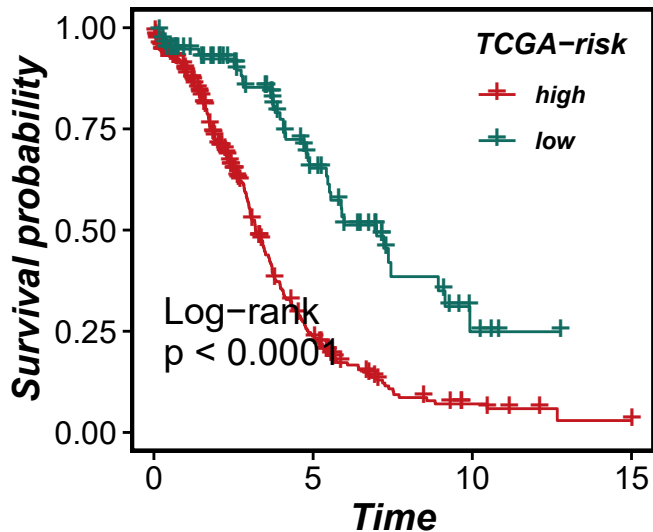

Supplement: Supplementary file 2 — Supporting Information 2 Figure S1: Kaplan–Meier survival curve showing LDLOCPS performance in the TCGA training cohort. [file MI-2025-6540537-s001.pdf]
